# Temporizing cast immobilization is a safe alternative to external fixation in ankle fracture-dislocation while posterior malleolar fragment size predicts loss of reduction: a case control study

**DOI:** 10.1186/s12891-022-05646-6

**Published:** 2022-07-22

**Authors:** Rene Gerlach, Andreas Toepfer, Matthijs Jacxsens, Viliam Zdravkovic, Primoz Potocnik

**Affiliations:** Department of Orthopaedics and Traumatology, Kantonsspital St. Gallen, 9007 St. Gallen, Switzerland

**Keywords:** Ankle fracture-dislocation, Malleolar fracture, Closed reduction, Cast immobilization, External fixator, Volkmann fragment

## Abstract

**Background:**

To determine if temporizing cast immobilization is a safe alternative to external fixator (ex-fix) in ankle fracture-dislocations with delayed surgery or moderate soft-tissue injury, we analysed the early complications and re-dislocation rates of cast immobilization in relation to ex-fix in patients sustaining these injuries.

**Methods:**

All skeletally mature patients with a closed ankle fracture-dislocation and a minimum 6-months follow-up treated between 2007 and 2017 were included. Baseline demographics, comorbidities, injury description, treatment history and complications were assessed.

**Results:**

In 160 patients (94 female; mean age 50 years) with 162 ankle fracture-dislocations, 35 underwent primary ex-fix and 127 temporizing cast immobilizations. Loss of reduction (LOR) was observed in 25 cases (19.7%) and 19 (15.0%) were converted to ex-fix. The rate of surgical site infections (ex-fix: 11.1% vs cast: 4.6%) and skin necrosis (ex-fix: 7.4% vs cast: 6.5%) did not differ significantly between groups (*p* = 0.122 and *p* = 0.825). Temporizing cast immobilization led to an on average 2.7 days earlier definite surgery and 5.0 days shorter hospitalization when compared to ex-fix (*p* < 0.001). Posterior malleolus fragment (PMF) size predicted LOR with ≥ 22.5% being the threshold for critical PMF-size (*p* < 0.001).

**Conclusion:**

Temporizing cast immobilization was a safe option for those ankle fracture-dislocations in which immediate definite treatment was not possible. Those temporized in a cast underwent definite fixation earlier than those with a fix-ex and had a complication rate no worse than the ex-fix patients. PMF-size was an important predictor for LOR. Primary ex-fix seems appropriate for those with ≥ 22.5% PMF-size.

**Trial registration:**

The study does not meet the criteria of a prospective, clinical trial. There was no registration.

## Background

Ankle fractures are among the most common fractures in adults with an incidence currently given as 71 – 200 per 100,000 person-years [1–8]. The incidence of these fractures have substantially risen over the last decades, in particular among elderly women who demonstrate more complex fracture patterns compared with other demographic groups [4, 5, 7, 9, 10]. Between 11 to 64% of all ankle fractures are classified as moderately to severely dislocated [2, 11–13], with fracture-dislocation and complex fracture patterns (i.e. bi- and trimalleolar fractures) demonstrating worse clinical outcomes compared with simple fractures [14, 15].

Open reduction and internal fixation (ORIF) is considered the mainstay of treatment for unstable malleolar fractures [6, 7, 10–13, 16–24]. Although immediate ORIF is recommended in the acute setting [7, 25], concomitant soft-tissue injuries can compromise long-term clinical outcome due to the increased risk of complications [18, 19]. To reduce the risk of soft-tissue problems, immediate reduction is mandatory while ORIF can be postponed until the swelling has subsided and the soft-tissues have consolidated [10, 16, 21, 26, 27]. Whereas fracture-dislocations with critical compromised soft-tissues (e.g., compartment syndrome, open fractures, skin blisters) are generally managed with an ankle-spanning external fixator (ex-fix) [28], the type of temporary fixation in those fracture-dislocation cases, in which the ideal time window for early ORIF has been missed and/or moderate to severe soft-tissue injury prohibit immediate ORIF is more open to debate. Besides ex-fix, also temporary immobilization in a splint or cast has been proposed as an alternative.

An ex-fix achieves reduction and stable fixation through axial traction and the resulting ligamentotaxis. Apart from the risk of pin tract infection, this type of fixation necessitates a staged additional surgery [29]. While a cast can provide a sufficient retention in a non-invasive manner, the retention is achieved by external pressure which carries the risk of additional soft-tissue damage and makes soft-tissue monitoring more difficult. With subsiding edema and decreasing swelling, the fit of the cast is reduced with the subsequent risk of losing the reduction over time [13, 23].

Given the limited evidence on the use of a temporizing cast, we sought to determine whether and for whom a cast immobilization is a safe alternative to temporary ex-fix in closed ankle fracture-dislocations. The aim of this study was to compare the short-term outcomes between ex-fix and plaster cast immobilization. Additionally, it was attempted to identify predictors for loss of reduction. It was hypothesized that both methods can achieve comparable short-term outcomes in those fracture-dislocation cases in which ORIF was delayed and/or moderate to severe soft-tissue injury prohibited immediate ORIF. The primary outcome were short-term soft-tissue-related complications. Secondary outcomes included loss of reduction, conversion to ex-fix, time to surgery and time to discharge.

## Methods

### Patient selection

The institutional database of our level-1 trauma center was analysed retrospectively for all patients with ankle dislocation-fractures treated surgically between 2007 and 2017. All skeletally mature patients with an ankle dislocation-fracture as a mono-trauma and a minimum 6-months follow-up were included. Skeletally mature was defined by a closed epiphysis on ap and lateral X-rays. Ankle dislocation-fractures was defined as ≥ 50% subluxation of the talus relative to the tibia in one of both planes on the X-ray or when a joint reduction maneuver was successfully performed before the X-ray was taken [30]. Excluded were those with incomplete records, open fractures, immediate definitive ORIF or treated nonoperatively.

### Demographics and outcomes

**Fig. 1:**
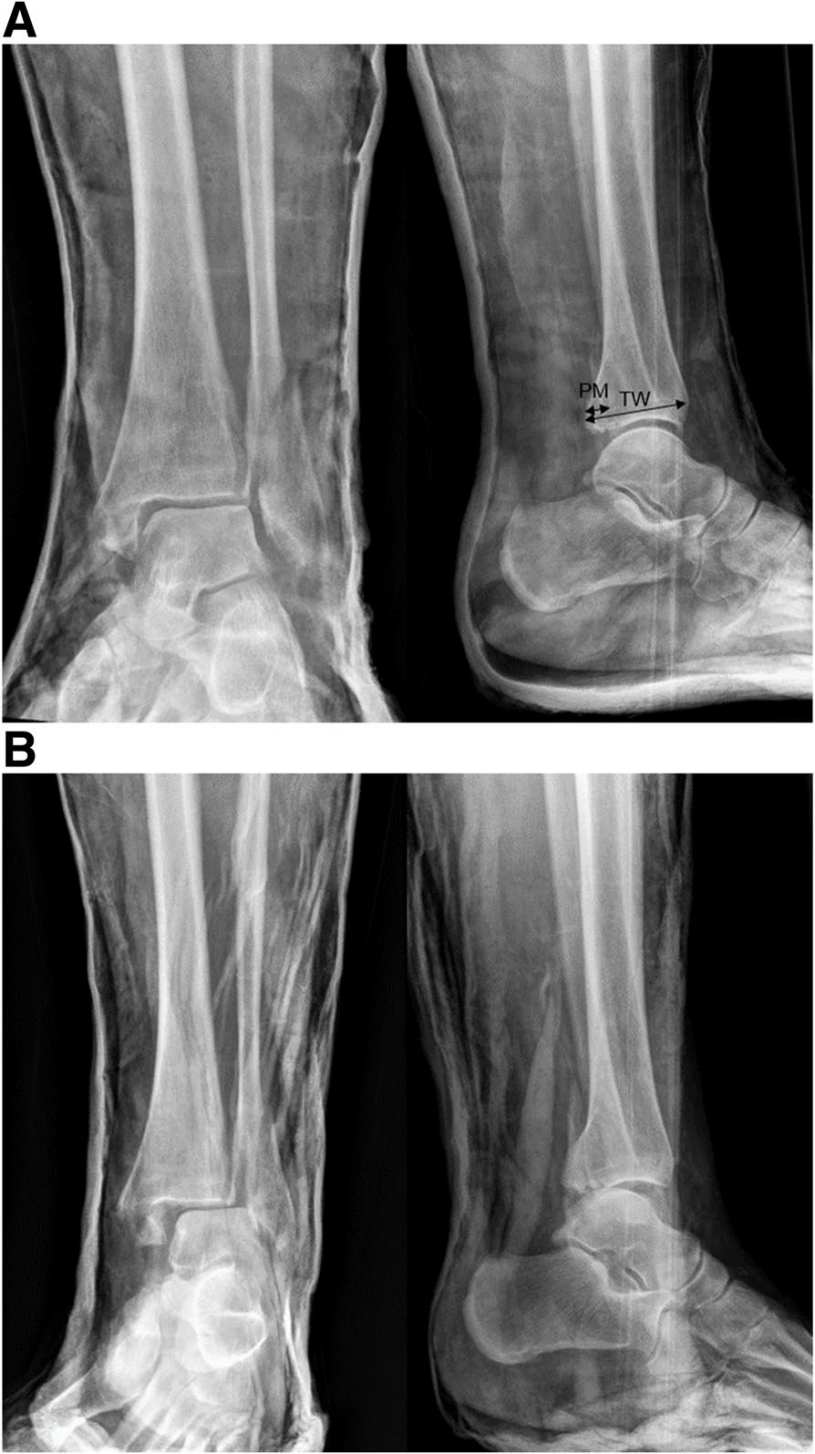
**A** Anteroposterior and lateral radiograph of a reduced trimalleolar ankle fracture-dislocation temporized in a plaster cast. Measurements were acquired at the level of the epiphyseal scar. The ratio between the width of the posterior malleolar fragment (PM) and the tibial width (TW) defined the posterior malleolar fragment size. In the presented case, the posterior malleolar fragment size was 27%. **B** Subsequent radiographs demonstrated a loss of reduction

All records of the eligible patients were reviewed to determine baseline demographics (age, gender, smoking, height, weight, BMI), comorbidities (diabetes, steroid use, anticoagulation use, ASA-score), injury description (side, fracture classification), treatment history (primary ex-fix, cast type, loss of reduction, conversion of treatment, time to conversion, time to surgery, time to discharge) and the evaluation of complications encountered during follow-up. Loss of reduction was defined by an incongruent tibiotalar joint with a dislocation of ≥ 5 mm in one of both planes on the X-ray. All fractures were classified independently by two of the authors using the Arbeitsgemeinschaft für Osteosynthesefrage/Orthopaedic Trauma Association (AO/OTA) classification system based on the available imaging (R.G. and P.P.) [31]. Although this classification system has a high interobserver reliability [32], a consensus between observers was made in case of doubt. Measurements were acquired at the level of the epiphyseal scar on the lateral radiographs. The ratio between the width of the posterior malleolar fragment and the tibial width defined the posterior malleolar fragment size (Fig. 1). Surgical site infections (SSI) were defined according the criteria of the Centers for Disease Control and Prevention (CDC) [18, 19, 33]. A superficial SSI (sSSI) was defined as requiring only antibiotics whereas a deep SSI (dSSI) needed revision surgery. Complex regional pain syndrome I (CRPS-I) was defined using the Modified (Budapest) International Association for the Study of Pain Criteria [34].

### Treatment

Immediate closed fracture reduction was performed as an emergency procedure. It was aimed to perform ORIF within 6-8 h from the time of injury. If immediate surgery was not possible, ORIF was postponed until the soft-tissue had recovered. First, it was tried to retain the reduction with a below-the-knee univalved fiberglass cast or plaster of Paris slab along with plaster of Paris stirrup. If there was insufficient reduction in the subsequent X-ray, an ex-fix was applied. In the case of secondary dislocation, a further attempt with closed reduction was made. If unsuccessful or a re-dislocation occurred, conversion to ex-fix was indicated. Moreover, conversion to ex-fix was indicated when the soft-tissue envelope was compromised by fracture blisters or skin necrosis. Ankle-spanning ex-fix was performed using two half-pins in the tibial shaft and one calcaneal Steinmann pin to create a delta frame. Both patients with ex-fix and patients with a cast immobilization were hospitalized for monitoring and decongestive measures. All surgeries were performed by a board-certified trauma surgeon or by residents under the supervision of the attending trauma surgeon according to the AO/OTA principles [35].

After definitive ORIF, the ankle was immobilized in a neutral position in a walker or a cast with limited weight-bearing using crutches. Early mobilization was performed out of the walker/cast instructed by a physiotherapist. These restrictions were recommended for 6 – 12 weeks. Low molecular heparin was routinely administered for thromboembolic prophylaxis. Routine points in time for clinical and radiographic evaluation were set at 6, 12, 24 and 52 weeks.

### Statistical analysis

The data were analysed using R (R: A language and environment for statistical computing. R Foundation for Statistical Computing, Vienna, Austria. URL http://www.R-project.org/). For numerical data, the Welch two-sample t-test was used. For categorical data, Pearson's Chi-squared test and Fisher´s exact tests were used, as appropriate. Multiple regression analyses determined predictors of loss of reduction. Predictors were further analysed with a receiver operating characteristic (ROC) curve analysis and Youden index analysis to determine a threshold. Differences were considered to be statistically significant when *p* ≤ 0.05. A post-hoc power analysis was completed based on the incidence rates in case statistical significance was lacking.

## Results

**Fig. 2:**
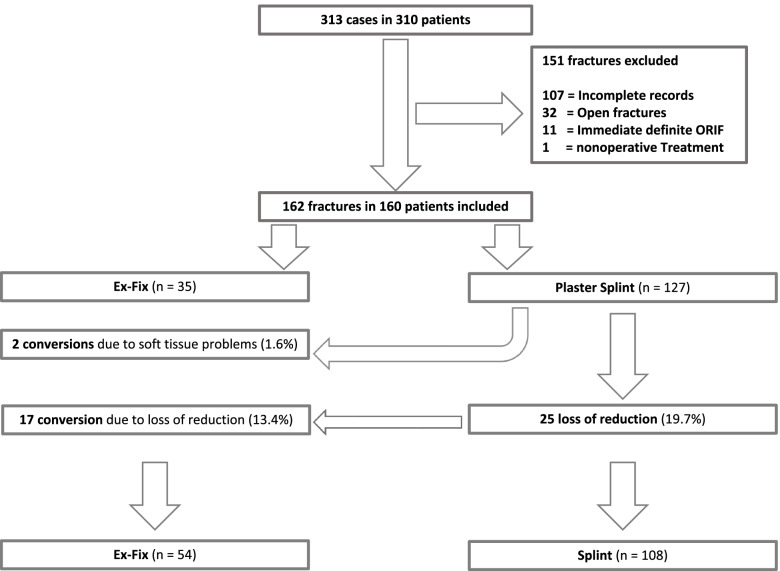
Flow chart of inclusion in the final external fixator (Ex-fix) group or plaster cast group

A consecutive series of 310 patients with 313 ankle fracture-dislocations met the inclusion criteria. A total of 151 cases were excluded, resulting in a study cohort of 160 patients with 162 ankle fracture-dislocations (Fig. 2). The cohort included 68 men and 94 women with a mean age of 50 years (range 20 to 87 years). Bimalleolar fractures occurred in 29.6% and trimalleolar fractures accounted in 70.4% of the cases. A lateral malleolar fracture type Danis-Weber B was seen in the majority of cases (74%) (Table 1). A posterior malleolar fracture was present in 53.1% of the cases.

**Table 1:** AO/OTA classification N (%)

Type	A	B	C
1	0	0	13 (8.0%)
2	2 (1.3%)	36 (22.2%)	23 (14.2%)
3	0	85 (52.5%)	3 (1.9%)
Total: 162	2 (1.2%)	121 (74.7%)	39 (24.1%)

Of the 162 fracture-dislocations, primary ex-fix was performed in 35 cases and 127 cases had a cast immobilization (mean age 50 years, 61 female). Of the latter, a total of 95 cases (74.8%) were immobilized with a fiberglass cast. A loss of reduction after temporizing cast was observed in 25 cases (19.7%) within a mean of 3 days (0–11 days): 17 of these cases were converted to ex-fix (13.3%); 7 cases were re-reduced in a cast (5.5%); in 1 case the dislocation was accepted and no action was required (0.8%). Another 2 cases were converted from temporizing cast immobilization to ex-fix because of a compromised soft-tissue envelope. Thus, 19 cases were converted to ex-fix (15.0%), resulting in 54 cases in the ex-fix group and 108 in the cast group (Fig. 2).

Baseline characteristics were comparable between ex-fix and cast group for the majority of variables (Table 2). Right side, smoking and osteoporosis skewed towards the ex-fix group (*p* < 0.047). No difference in fracture type was found between the ex-fix and the plaster cast group (*p* = 0.068). Ex-fix-cases underwent definite ORIF on average 2.7 days later and were hospitalized on average 5.0 days longer when compared to patients with temporizing cast immobilization (*p* < 0.001) (Table 3).

**Table 2:** Baseline Characteristics

		Ex-Fix (*n* = 54)	Plaster cast (*n* = 108)	*p*
Mean age, years (range)		53.3 (20.9–87.0)	50.1 (15.5–83.3)	0.252^+^
Gender, N	male/female	21/33	47/61	0.694^*^
Side, N (%)	right/left	34/20 (63%/37%)	40/68 (37%/63%)	0.003^*^
Mean BMI, kg/m^2^ (range)		26.1 (17.3–41.2)	26.8 (17.3–41.0)	0.31^+^
Diabetes, N (%)		5 (9.3%)	6 (5.6%)	0.581^*^
Smoking, N (%)		18 (33.3%)	24 (22.2%)	0.047^*^
Osteoporosis, N (%)		7 (13.0%)	3 (2.8%)	0.028^*^
ASA class, N (%)	1	21 (38.9%)	53 (49.1%)	
	2	28 (51.8%)	49 (45.4%)	
	3	5 (9.3%)	6 (5.5%)	0.292^*^
Steroid use, N (%)		3 (5.6%)	3 (2.7%)	0.659^&^
Anticoagulation, N (%)		2 (3.7%)	2 (1.9%)	0.858^&^
Mean Follow-up, mths (range)		16.9 (6–63)	15.4 (6–63)	0.392^+^

^*^Chi-Square test

^+^T test

^&^Fischer test

**Table 3:** Results

	Ex Fix (*n* = 54)	Plaster cast (*n* = 108)	*p*
time to surgery (days), mean (range)	9.1 (4–21)	6.4 (2–16)	< 0.001
time to discharge (days), mean (range)	18.1 (9–34)	13.1 (5–33)	< 0.001
SSI, N (%)	6 (11.1%)	5 (4.6%)	0.122
sSSI, N (%)	3 (5.6%)	2 (1.9%)	
dSSI, N (%)	3 (5.6%)	3 (2.8%)	
Skin necrosis, N (%)	4 (7.4%)	7 (6.5%)	0.825
CRPS-I, N (%)	5 (9.3%)	3 (2.7%)	0.073

*SSI* surgical site infection, *sSSI* superficial surgical site infection, *dSSI* deep surgical site infection, *CRPS* complex regional pain syndrome

At least one complication was seen in 41 patients (25.3%). Soft-tissue complications were seen in 22 patients (13.6%) with 11 (6.8%) patients having a SSI and 11 (6.8%) presenting skin necrosis. CRPS was diagnosed in 8 patients (4.9%) (Table 3 & 4). The rate for SSI and skin necrosis did not differ significantly between ex-fix and cast immobilization (*p* = 0.122 and *p* = 0.825). A tendency toward a lower CRPS-I-rate was seen in favor of the cast group (*p* = 0.073). A post-hoc power analysis showed a needed sample size of 536, 25064 and 420 patients to detect a difference in the rate of SSI, skin necrosis and CRPS-I, respectively, using a 95% confidence interval and a power of 80%.

**Table 4:** .

Complications	N (%)
Surgical site infection	11 (6.8%)
Superficial surgical site infection	5 (3.1%)
Deep surgical site infection	6 (3.7%)
Skin necrosis	11 (6.8%)
Complex regional pain syndrome I	8 (4.9%)
Deep venous thrombosis	1 (0.6%)
Posterior Tendon irritation by callus	2 (1.2%)
Peroneal tendon irritation	1 (0.6%)
Flexor hallucis longus tendon partial tear	1 (0.6%)
Achilles tendon shortening with Arthrofibrosis	1 (0.6%)
Tibiofibular syndesmosis malreduction	4 (2.5%)
Intraarticular screw placement	1 (0.6%)
Malunion	1 (0.6%)
Delayed-/Non-union	3 (1.8%)

**Fig. 3:**
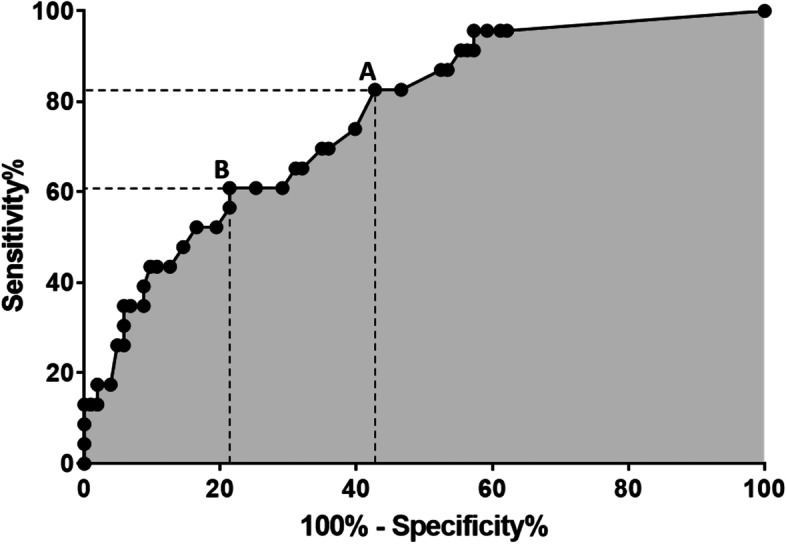
Receiver operating characteristic (ROC) curve analysis demonstrated an area under the curve of 0.766 (*p* < 0.001) and identified two thresholds for posterior malleolar fragment size to predict loss of reduction in a cast with the same tradeoff between sensitivity and specificity. A threshold of 13.5% (**A**) resulted in a sensitivity of 83% and a specificity of 57%. A threshold of 22.5% (**B**) was associated with a sensitivity of 61% and a specificity of 79%

Multiple regression analyses revealed posterior malleolar fragment size as an independent risk factor for loss of reduction after cast immobilization (*p* < 0.001), whereas age, gender, side, BMI, co-morbidities, osteoporosis, fracture classification, the presence of a posterior malleolar fragment and cast type were not associated with re-dislocation (*p* > 0.085). ROC-analysis of posterior malleolar fragment size resulted in an area under the curve of 0.766 (*p* < 0.001) (Fig. 3).

## Discussion

The findings of this study demonstrate that within the presented cohort, a temporizing cast immobilization was associated with no worse results compared to ex-fix. A trend toward a less favorable rate of soft-tissue-related complications was seen for ex-fix. Those patients temporized with a cast underwent definite fixation earlier than the patients with ex-fix, which resulted in a shorter length of stay. An important finding of this study was that posterior malleolar fragment size was the sole predictor of loss of reduction of all assessed variables. Therefore, casting of patients with a critical posterior malleolar fragment size must be discussed.

Soft-tissue problems associated with temporizing cast immobilization were comparable with the rates reported by other authors with SSI ranging between 1 to 20% and skin necrosis rate ranging between 4.9 to 8.9% [6, 12, 17, 24, 25, 29, 30]. Wawrose et al. compared 28 patients with temporizing plaster splint immobilization to 28 patients with ex-fix following ankle fracture dislocation [30]. The authors found that splint immobilization was associated with a high skin necrosis rate of 17.8% when compared to a temporizing ex-fix which yielded 0% skin necrosis. The rather high necrosis rate of 17.8% and sSSI rate of 17.8% in the splint group and the absence of complications in the ex-fix group favorized the use of ex-fix as a temporizing fixation following ankle dislocation fractures. These remarkably good results following ex-fix could not be reproduced at our institution; yet, our necrosis rate within the cast group was substantially less. These differences between this study and our study might be a result of the high re-dislocation rate of 50% in the splint group seen by Wawrose et al.

Loss of reduction was contributed to the type of cast in the study by Baker et al. [36]. A 50% (11/22 cases) loss of reduction was noticed when temporizing immobilization was performed with a plaster splint, whereas no re-dislocation was seen in the bivalved fiberglass cast group (0/17). In our study, the type of cast was not a predictor for loss of reduction. Only size of the posterior malleolar fragment was highly associated with loss of reduction. Although size of the posterior malleolar fragment does not correlate with clinical and radiological outcomes after ORIF [37], our findings support the idea that the posterior malleolus is an important indicator for fracture stability of the ankle. Given this result, the substantial difference between our moderate re-dislocation rate of 19.8% within the cast group and an unacceptable high re-dislocation rate of 50% reported by Wawrose et al. might be attributed to differences in posterior malleolar fragment sizes between the study groups [30]. It must be noted that, in contrast to our study, patients included in the study by Wawrose et al. were discharged after splinting, which makes strict monitoring of the ankle and a prompt reaction to soft-tissue problems difficult. Moreover, the lack of control of patients’ behavior at home might lead to noncompliance with patients walking on the cast.

As stated by Mittlmeier et al., it remains a question of definition to distinguish fractures of the posterior pilon from ankle (luxation) fractures [38]. According to the classification of Bartonícek and Rammelt, type 1 and 2 fractures are caused by a combination of tensile, compressive, and shear forces and thus are most likely to result from capsulo-ligamentous bony avulsion mechanisms whereas type 3 and 4 fractures with a large articular component show morphologic overlap with partial pilon fractures [39, 40]. This is consistent with the observations of other authors [41] without a clear consensus regarding delineation [42].

In another retrospective study Buyukkuscu et al. showed a higher rate of reduction loss and skin necrosis in the splint group (*n* = 69) compared to the external fixator group (*n* = 48) [43]. The time to surgery was shorter in the external fixator group. This difference may be explained by inclusion of patients with poor soft tissue conditions only.

Regarding predictors, a ROC-analysis identified two thresholds for posterior malleolar fragment size to predict loss of reduction in a cast demonstrating the same tradeoff between sensitivity and specificity (Fig. 3). Whereas a 13.5% threshold would overcome a re-dislocation in a temporizing cast in the majority of cases, a substantial percentage of patients would be treated with an additional surgery that was not necessary due to the false-positive rate associated with a specificity of 57%. Since our results regarding complications with cast immobilization were promising, we prefer a more conservative approach. Therefore, the 22.5% threshold, which allows for some degree of re-dislocation but overcomes surgical overtreatment, was preferred as a cutoff for critical posterior malleolar fragment size to define ankle fracture-dislocations in which a primary temporizing ex-fix seems appropriate.

Our study is limited by its retrospective nature. The slightly skewed distribution between groups and the limited power are exemplary for this. The latter highlights the challenges associated with a monocentric study, even in a level-1 trauma center, in which small differences in complication rates are seen, needing a larger sample size to provide a sufficient power. Patients were treated by several surgeons, since this retrospective analysis covered 10 years of treatment in a teaching hospital. At the time, no clear indication to do a primary ex-fix was defined and depended on surgeon´s decision-making. This imposes a further selection bias of the study cohort. This bias in selecting patients for primary external fixation may partly explain why a difference of 5 days in average hospital stay was found between the 2 groups. Because of the multiple variables that were needed for the analyses, a substantial number of cases had to be excluded due to incomplete data. A prospective assessment would have increased the sample size by assessing those variables in a standardized fashion. The short-term follow-up of a minimum of 6 months prohibited us to make any conclusions on long-term outcomes on the initial use of a cast versus ex-fix for ankle fracture-dislocation management. Although the majority of cases underwent a CT scan of the ankle, the size of the posterior malleolar fragment was only assessed on lateral radiographs. This measurement might be influenced by malrotated views, as commonly seen in the acute setting. Yet, radiographs are the primary images acquired in the acute assessment of an ankle fracture and hence makes the results of this study more applicable for quick decision making.

## Conclusions

Temporizing cast immobilization was a safe and viable option for those ankle fracture-dislocations in which early ORIF was not possible. Those temporized with a cast underwent definite fixation earlier than the patients with ex-fix, which resulted in a shorter length of stay. Posterior malleolar fragment size was an important predictor for loss of reduction in a cast, with 22.5% being identified as the cutoff for critical posterior fragment size. Therefore, a primary temporizing ex-fix in those patients with a posterior malleolar fragment approximating one fourth of the distal tibial articular surface seems appropriate.

## Data Availability

The datasets used and analysed during the current study are available from the corresponding author on reasonable request.

## Abbreviations

AO/OTA: Arbeitsgemeinschaft für Osteosynthesefrage/Orthopaedic Trauma Association
ASA: American Society of Anesthesiologists
BMI: Body Mass Index
CDC: Centers for Disease Control and Prevention
CRPS-I: Complex regional pain syndrome I
dSSI: Deep surgical site infections
Ex-fix: External fixator
LOR: Loss of reduction
ORIF: Open reduction and internal fixation
PMF: Posterior malleolus fragment
ROC: Receiver operating characteristic
SSI: Surgical site infections
sSSI: Superficial surgical site infections
